# An Extraordinary Case in Practice

**Published:** 1885-08

**Authors:** R. T. Knox

**Affiliations:** Gonzales, Texas


### AN EXTRAORDINARY CASE IN PRACTICE.

*By R. T. Knox, M. D., Gonzales, Texas.*

For Daniel's Medical Journal.

I WAS called to see Mrs. D., some mile or more from Gonzales, on the evening of May 10th, 1885. Found her in labor,—claimed to be within a few days of full time. She was aged about 22 years,—this being her second confinement. She informed me she had worked in her garden in the morning, after which she visited her father's home, some nine miles, where I found her. Had no notion of sickness until after her arrival there some hours. General health said to be good.

On examination, I found the first stage of labor well established, and a substance of indefinite character presenting itself, composed of bones and muscular substance, all flattened or cup shaped ; the pains being regular, very soon expelled a dead fœtus, having the appearance of being some 4½ or 5 months formation, and having a dried or emaciated look, with right side of head cupped as though some round substance had long rested upon it. Further search revealed the fact of another child presenting, head first, which was born within the next half hour, fully matured, but vitality very weak, feeble cry, and which, after being placed to the breast, nursed but poorly, and did not improve in any particular, but died on the fourth day after birth. The mother did well in every way ; no fever, septic, or otherwise ; only slight trouble with her breasts. She was up and at her usual duties in due time.

I preserved the dead fœtus, and showed it to several physicians and others. There was one placenta, small, and not of a healthy cast ; the one cord of living child full large, the other the reverse.

I report this case, as it was one new to me in my practice of many years, and at first hard to define what I had found.

The inquiry often occurs to me, why so little or no disturbance in the healthy economy of the mother in such a peculiar condition.

